# Tailoring Pt-Based Organometallic Porous Network on Ag(111): A Model System for “Host-Guest” Chemistry

**DOI:** 10.1021/acsnanoscienceau.5c00124

**Published:** 2025-11-07

**Authors:** Vanessa Carreño-Diaz, Alisson Ceccatto, Eidsa Brenda da Costa Ferreira, Majid Shaker, Hans-Peter Steinrück, Abner de Siervo

**Affiliations:** † Institute of Physics Gleb Wataghin, 28132 University of Campinas, 13083-859 Campinas, Brazil; ‡ Lehrstuhl für Physikalische Chemie II, 9171 Friedrich-Alexander-Universität Erlangen-Nurnberg, 91058 Erlangen, Germany

**Keywords:** on-surface synthesis, porous network, single-atom catalyst, scanning tunneling microscopy, SMONs, host−guest

## Abstract

Metal–organic frameworks (MOFs) have proven to be versatile platforms for anchoring individual metal atoms, which can act as single-atom catalysts. Due to their well-defined geometric and electronic structure, high porosity, and adjustable pore size, MOFs can modulate the catalytic performance of anchored individual atoms. In this work, we explored the surface-assisted synthesis of 2D surface metal–organic networks (SMONs) of 1,3,5-tris­[4-(pyridine)-[1,1’-biphenyl]­benzene] (TPyPPB) coordinated with Pt atoms on Ag(111) by using scanning tunneling microscopy at room temperature. The Pt deposition was performed in two routes: (i) by using the dichloro-(1,10-phenanthroline)-platinum­(II) (Cl_2_PhPt) or (ii) by direct deposition of Pt atoms. Using Cl_2_PhPt as a Pt source and applying various annealing sequences at a temperature of 400 K, a long-range hexagonal SMONs is obtained. After the dechlorination of the Cl_2_PhPt molecule, individual Pt atoms establish quadruple coordination with two N atoms at the pyridyl end groups of the TPyPPB molecule and two Cl atoms. These pores have efficiently induced the formation of large molecules that behave like rotors. Such a system has the potential to open new frontiers and shed light on a better understanding of the physical-chemistry mechanisms involved in “host-guest” chemistry.

## Graphical abstract

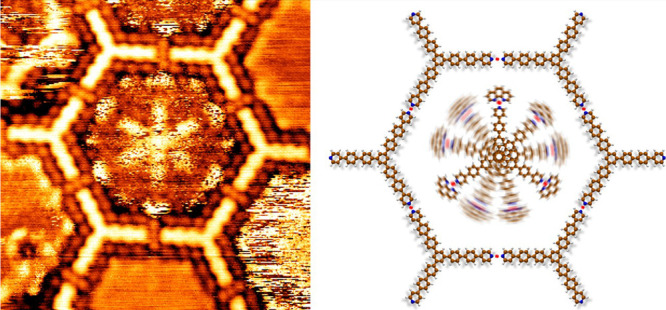

## Introduction

Single-atom catalysts have attracted considerable attention in the last two decades due to their selectivity and efficiency in heterogeneous catalysis.^1–3^ They have been used in several applications, from fuel production to fine chemicals synthesis.^4–8^ A key challenge of most conventional catalysts is that they can be formed by metallic nanostructures consisting of groups of metallic atoms organized in particles of different sizes and shapes.^1,9^ Typically, under-coordinated metal atoms located on surfaces or in low-coordination environments are the most reactive. However, such sites may be scarce in larger particles or poorly distributed in irregular nanostructures. As particle size decreases, the proportion of surface atoms increases relative to the volume, enhancing the catalytic activity per metal atom.^10^ Nevertheless, this effect is limited, as smaller particles exhibit higher surface free energy, which promotes the aggregation of metal atoms.^11–14^ This heterogeneity in active site distribution poses a significant challenge, complicating the optimization of catalytic efficiency and potentially leading to unwanted byproducts in complex reactions. Furthermore, nearly half of conventional catalysts rely on precious metals (e.g., Pd, Pt, Rh), which are rare on Earth, making high-performance commercial catalysts costly.

Single-atom (SA) systems can achieve uniform distribution, high catalytic efficiency, and reaction selectivity.^15,16^ Single-atom catalysts (SACs) are defined as supported metal catalysts in which metals are dispersed as individual atoms, either uniformly spaced on the surface or anchored within the framework of the support, such as oxides, carbon-based materials, metal sulfides, and MOFs.^17–19^ The development of stable SACs requires the synthesis of supports that interact with single atoms, avoiding their aggregation. This atomic dispersion makes MOFs an ideal SACs model system for studying and understanding heterogeneous catalysis at the atomic and molecular levels.^20–22^ MOFs are more traditionally obtained in the three-dimensional forms via coordination bonds, forming rigid and porous crystalline structures with well-defined cavities and channels. 3D MOFs are usually grown via solution-based solvothermal methods, often producing bulk crystalline powders. It can also form thin films using layer-by-layer growth methods; however, the structural control is more challenging at the atomic level. In contrast, the 2D-MOFs, often called surface metal–organic networks (SMON), are synthesized via surface-assisted growth (e.g., on metal substrates or at liquid–air interfaces). 2D-MOFs possess controlled growth on surfaces, enabling atomic-scale imaging, such as STM and AFM, allowing direct visualization of atomic and molecular processes, such as metal insertion, ligand exchange, or defect formation. 2D-MOFs are useful for electrocatalysis, sensing, membrane separation, and interface studies. The exposed metal sites and controllable orientation make them excellent model catalysts, acting as a perfect model system to improve the basic understanding of the physical-chemical mechanisms involved in different types of reactions.^23–25^

Recent developments in two-dimensional materials have significantly improved both the performance and the uniform activity of SAC.^26^ In particular, MOFs have demonstrated outstanding potential as SAC supports for catalytic processes due to their large surface areas and well-defined pore structures, enabling a deeper understanding of the relationship between structural features and catalytic performance. Studies have shown that carbon-based supported SACs can be readily synthesized from MOF precursors through simple pyrolysis.^27,28^ For example, carbonization of MOFs containing nitrogen species results in nitrogen-doped porous carbon materials that provide abundant anchor sites for immobilizing individual metal atoms, allowing the formation of stable SACs.^29,30^

The combination of MOFs/SAC has been utilized, e.g., as a photocatalyst to drive various reactions, including pollutant degradation,^31^ water splitting for hydrogen production,^32^ and CO_2_ reduction.^33^ The effectiveness of MOFs/SAC is attributed to their tunable structural properties, which optimize light absorption, reduce HOMO–LUMO energy gap, enhance electron–hole separation, uniformly distribute catalytic active sites, and improve their accessibility for reactants.^34^ Single-atom photocatalysts maximize atomic efficiency, as each isolated metal atom serves as an active site, unlike traditional photocatalysts based on nanoparticles or larger structures, where not all atoms actively participate in the catalytic process.^35–38^ The unique electronic structure of single atoms enables more precise control over photocatalytic reactions. The under-coordinated nature of these sites promotes specific interactions with reactants and photogenerated species, influencing reaction pathways.^16,39,40^ Furthermore, porous MOFs/SACs are of great interest due to their unique advantages of tunable pore sizes, which can be utilized in various applications, including “host-guest” chemistry,^41^ molecular separation, molecular detection, and sensing.^42,43^ For instance, by trapping molecules and atoms in a confined space, a specific reaction is promoted that favors the formation of macromolecules and clusters with a particular shape and size, and emergent properties.^44–48^

In this study, we have employed scanning tunneling microscopy (STM) to investigate the coordination process of the molecular precursor 1,3,5-tris­[4-(pyridin-4-yl)-1,1′-biphenyl]­benzene (TPyPPB) (Figure 1a) for the formation of Pt-based surface metal–organic networks (Pt-SMONs) on a Ag(111) surface. A 2D version of a Pt-based MOF can serve as a model system to better understand the formation and stabilization mechanisms in MOFs/SAC, as well as allow for direct visualization of molecular confinement and the formation of molecules with specific size and shape inside the pore, in the so-called host–guest chemistry. Two approaches were employed to introduce the coordinating metal atom into the system. In the first approach, Pt atoms were directly deposited on the Ag(111) surface containing TPyPPB, resulting in clusters of several sizes that hindered the formation of long-range coordinated Pt-SMONs due to the high surface energy of Pt atoms. The second approach involved codepositing TPyPPB and the molecular precursor dichloro­(1,10-phenanthroline)-platinum­(II) (Cl_2_PhPt) (Figure 1b) as a Pt atom source. Upon annealing to moderate temperatures (∼400 K), chemical transformations of Cl_2_PhPt occur. After dehalogenation, the Cl_2_PhPt precursor transforms into the intermediate complex PhPt, which anchors at the end of the pyridyl group of TPyPPB molecules, leading to various ordered patterns. The Cl atoms dissociated from the Cl_2_PhPt precursor migrate to the periphery of TPyPPB molecules. Further annealing produces a complete transformation of the PhPt intermediate, providing Pt atoms that react with Cl atoms to form the PtCl_2_ units that stabilize the TPyPPB molecules via metal coordination between Pt and the pyridyl group (N–Pt–N). We also discuss in detail the role of Cl atoms in stabilizing the Pt-SMONs. This organometallic coordination yields an almost perfect hexagonal pore network on the surface. It particularly forms large pores that can accommodate macromolecules of approximately 5.0 nm in diameter, which STM directly imaged.

**Figure 1:**
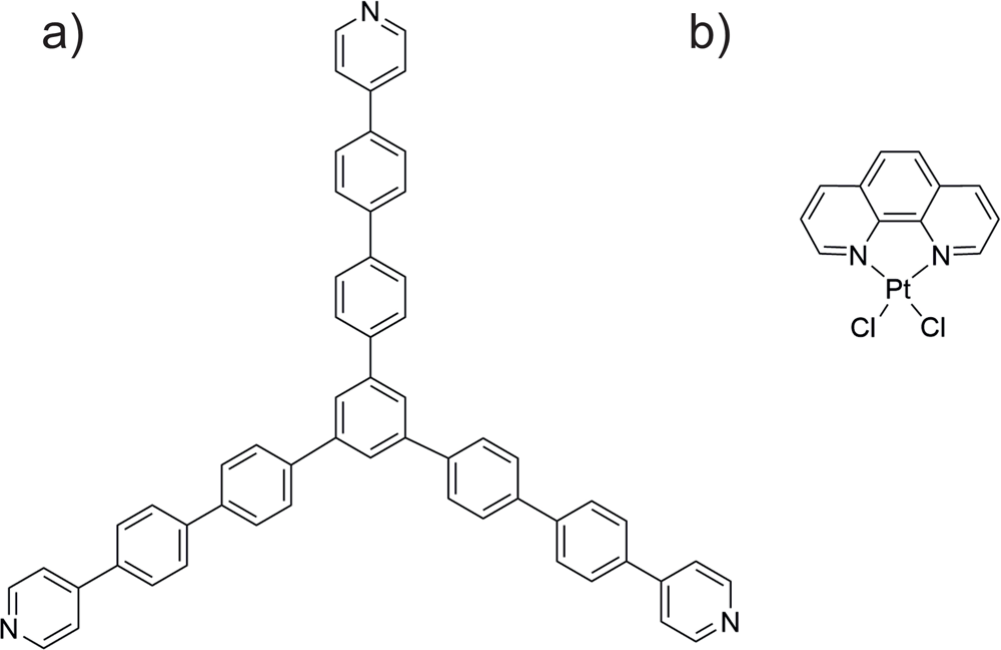
Molecular structures for the (a) TPyPPB and (b) Cl_2_PhPt precursors.

## Materials and Methods

The experiments were performed at UNICAMP in Campinas (Brazil) using a UHV surface science apparatus. The experimental setup consists of two interconnected chambers (STM and XPS). The X-ray photoelectron spectroscopy (XPS) chamber, used for sample preparation, has a base pressure in the low 10^–10^ mbar range. It is equipped with a SPECS Phoibos 150 high-resolution hemispherical analyzer with multichannel detectors and a dual Al/Mg K_α_ X-ray source. It also includes a five-axis manipulator equipped with sample heating capabilities (room temperature (RT)—1500 K), as well as e-beam and Knudsen cell evaporators, along with sample cleaning facilities. The base pressure in the scanning tunneling microscopy (STM) chamber was in the intermediate 10^–11^ mbar range. The STM measurements were performed using a SPECS Aarhus 150 microscope, operated with a SPECS SPC 260 controller, and a bias voltage was applied to the sample. The measurements were performed in constant tunneling current mode using a W tip cleaned in situ by Ar^+^ sputtering. The Ag(111) single crystal was prepared by cycles of Ar^+^ sputtering (600 V; 7 μA cm^–2^) followed by annealing at 773 K for 15–30 min with a slow heating/cooling ramp (0.3 K/s). TPyPPB molecules were purchased from ET Chem Extension, while Cl_2_PhPt molecules were purchased from Sigma-Aldrich. The molecular precursors were deposited using a homemade 3-fold Knudsen cell evaporator, allowing for the independent evaporation of up to three molecular precursors at temperatures below 870 K. The TPyPPB and Cl_2_PhPt molecules were sublimated from a quartz crucible at 653 and 623 K, respectively. Pt 99.999% was evaporated from a 1 mm Pt-Rod using a Focus e-beam evaporator. The typical Pt deposition parameter was an e-beam voltage of 1000 V and an emission current of 19 mA, resulting in a Pt ion flux of approximately 12 nA, which was measured and maintained constant in the evaporator. All depositions were performed with the Ag(111) substrate held at RT and the coverage calibrated using STM images. All STM images were calibrated using a correction matrix obtained from the Ag(111) atomic resolution and analyzed using Gwyddion software.^49^

## Results and Discussion

### Coordination Route 1: TPyPPB + Pt Metal

In the first step, we have calibrated Pt deposition procedure. Pt was deposited onto the clean Ag(111) surface kept at RT using a pure Pt rod for 15 min, resulting in a submonolayer coverage, as shown in Figure 2a. In this image, we observed the formation of Pt islands, which appear as bright areas. Bunddhika et al.^50^ reported similar results, where the bright contrast exhibited heights of approximately (0.285 ± 0.014) nm. In the inverse Ag on Pt(111) system, Brune et al.^51^ also noted bright spots with a height of 0.29 nm, which they attributed to Ag dimers. The growth of Pt islands on Ag(111) can be described through a two-step mechanism. First, Pt atoms replace Ag atoms on the substrate via an exchange process. Second, the Pt atoms that have replaced the Ag ones act as nucleation sites for other Pt atoms, leading to the growth of incorporated 2D Pt islands.^50^

**Figure 2:**
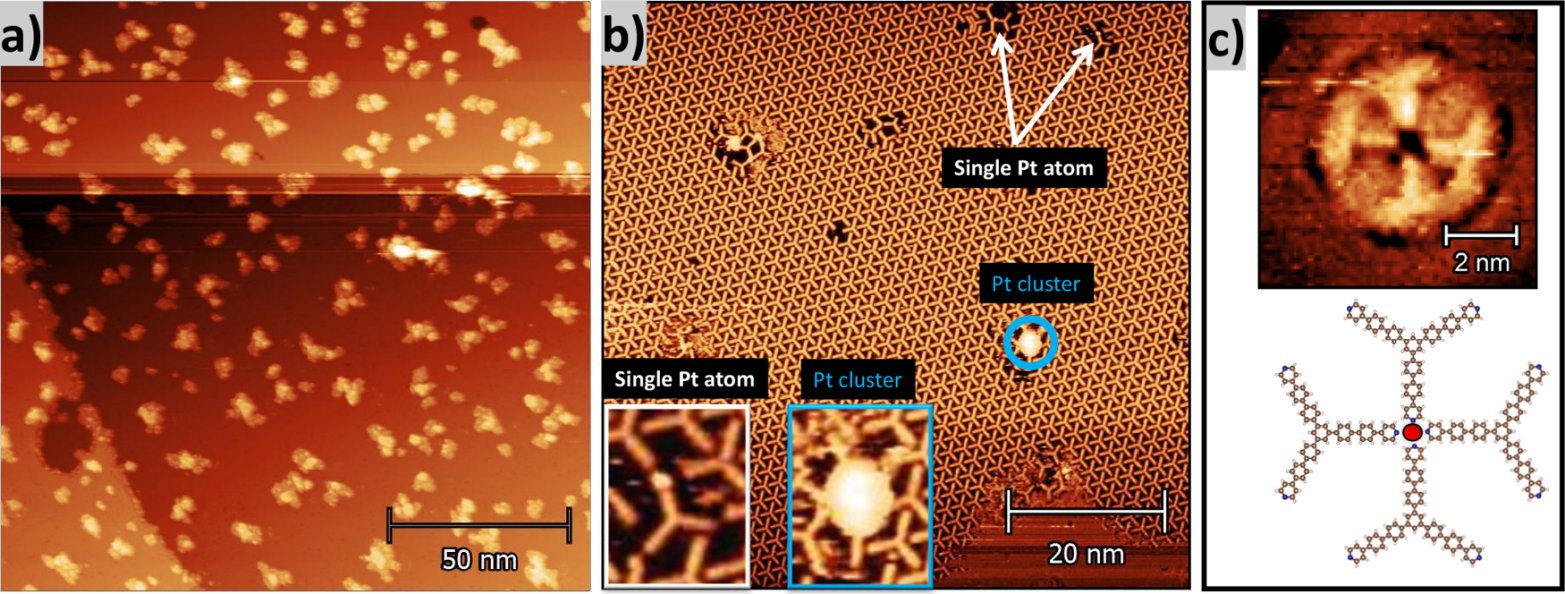
STM images. (a) Formation and distribution of Pt islands on the Ag(111) surface (*V*_*t*_ = −1489 mV, *I*_*t*_ = 270 pA). (b) Triangular packing between TPPyPPB molecules with some defects due to interactions with a single Pt atom (white arrows) or a Pt cluster (blue circle) (*V*_*t*_ = −857 mV, *I*_*t*_ = 660 pA). (c) Pt­(N_4_) coordination between 4 TPyPPB molecules and a single Pt atom (*V*_*t*_ = −911 mV, *I*_*t*_ = 410 pA).

A series of experiments was conducted to form two-dimensional Pt-based surface metal–organic networks (2D Pt-SMONs), consisting of the subsequent evaporation of the TPyPPB molecule and Pt onto the Ag(111) surface. In the first step, the TPyPPB molecule was deposited (in the submonolayer regime) on Ag(111), promoting the formation of extended islands of supramolecular networks. Ceccato et al.^52^ reported that when the TPyPPB molecule is deposited on the clean Ag(111) surface, a porous nanostructure is formed, characterized by regular triangular pores, which are stabilized by N···H hydrogen bonds between the TPyPPB molecules. Thereafter, 1 min of Pt (very low dose, ∼0.01 ML) was deposited and subsequently annealed at 400 K. The formation of a 2D porous supramolecular network was observed, as shown in Figure 2b. This network extends over tens of nanometers and exhibits a low density of defects.^52^ In the present study, the defects in the network are attributed to the coordination between the pyridyl end groups of the TPyPPB molecules and the Pt atoms. We observed two different coordination modes: one in the single atom regime, where the molecules are coordinated through a single Pt atom (which might be the case indicated by white arrows in Figure 2b), and where the TPyPPB molecules also coordinate with Pt clusters, and nanoparticles (indicated by a blue circle in Figure 2b). Through statistical analysis of tens of different STM images, we estimated that these Pt islands have lateral sizes ranging from 1.4 to 3.1 nm and a height of 0.18–0.39 nm. Another type of structure was consistently observed in the STM images, where TPyPPB molecules form a 4-fold coordination, which from STM images could be attributed to a single Pt atom via N–Pt–N bonds (PtN_4_) (Figure 2c). Nevertheless, from solely the STM images, it is not possible to completely exclude a multinuclear Pt coordination structure as reported in literature for other systems.^53^ In the case of a single Pt atom coordination, the results might be comparable with the work of Zuo et al.,^15^ where ultrathin 2D-MOFs were formed using Pt-metalated porphyrins, which also show N–Pt–N bonds (PtN_4_). The layers coordinated with individual Pt atoms at ultrahigh concentrations (namely PtSA-MNSs) were synthesized for highly efficient photocatalytic H_2_ evolution, using Pt­(II) tetrakis­(4-carboxyphenyl)­porphyrin (PtTCPP).

A second subsequent deposition of an additional 2 min was carried out in the same system to increase the concentration of Pt atoms (∼0.03 ML). Although the same supramolecular network of triangular pores was still observed in Figure 3a, pronounced changes of the system are also evident, with the formation of a new amorphous, short-range Pt-MOF as shown in Figure 3b. Figure 3c shows the TPyPPB molecules forming motifs with two- and 3-fold coordination, as highlighted by the blue and green circles, respectively. We assign this behavior to N–Pt coordination, since it is well-known from the literature that Ag adatoms do not coordinate with pyridyl and cyano end groups.^52,54–59^ Nevertheless, it is not possible to say solely from the STM images that these coordinations are made by single-Pt atoms. For instance, we can observe in Figure 3c that 2-fold coordination nodes are very bright, while 3-fold nodes are dim, which might also indicate these coordination nodes are formed by a few Pt atoms instead of a single Pt atom. For instance, Wang et al.,^53^ have demonstrated several examples where more than one atom frequently occurs in a multinuclear metal–organic coordination that contains metal-cluster nodes. Driven by the free energy,^60,61^ small noble metals (individual atoms or clusters) tend to aggregate into larger particles, which is why some molecules were also observed forming bonds with Pt clusters (yellow circle, Figure 3c). To decrease the formation of Pt islands, we performed an annealing step at 400 K. However, after this thermal treatment, dramatic changes were observed. The triangular pore network was destroyed, and Pt islands continued to form with a similar appearance, with lateral sizes ranging from 1.4 to 2.1 nm and heights of 0.3 nm as shown in Figure 3d. Moreover, we start to observe more frequently the formation of 3-fold coordinated molecules, which also might be an indication of stabilization by multinuclear metal–organic coordination.^53^ This new layer turned out to be much more disordered, with TPyPPB molecules almost entirely coordinating through Pt clusters of different sizes and shapes.

**Figure 3:**
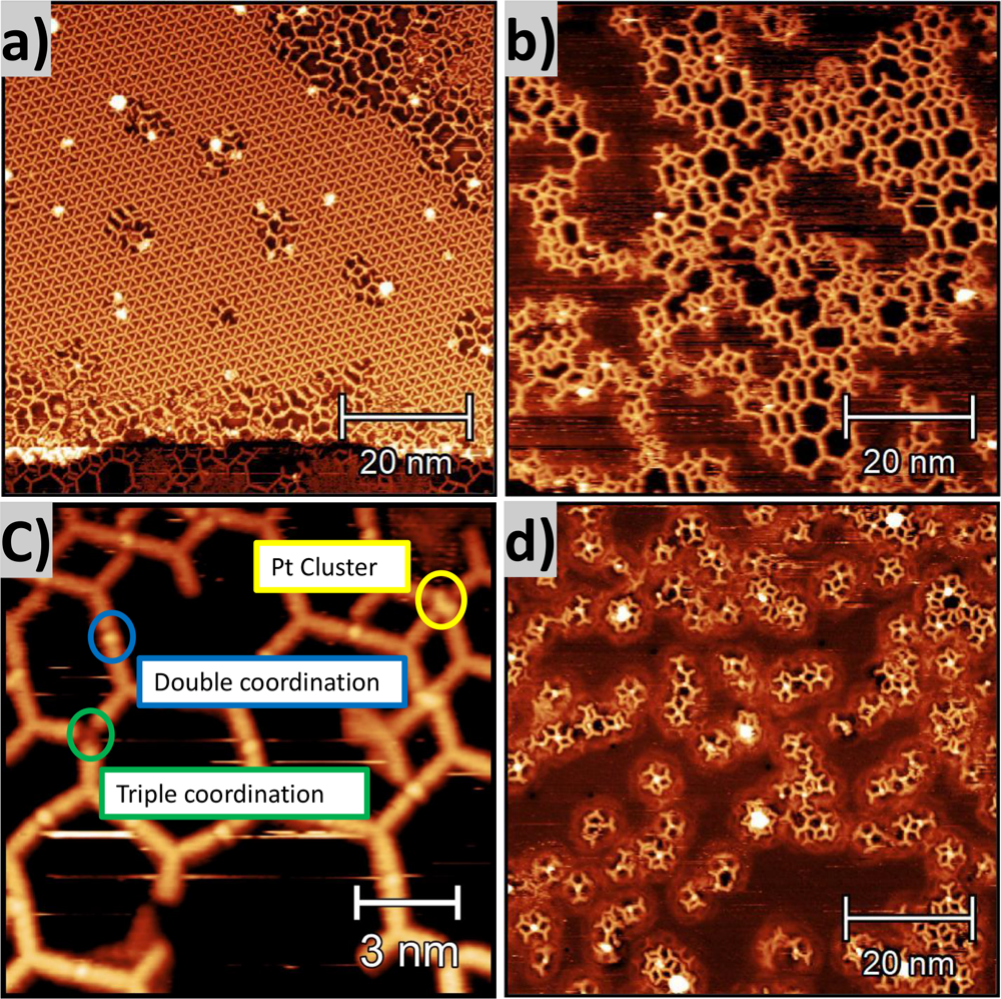
STM images (a) mixture between two phases: triangular packing and formation of Pt-SMON (*V*_*t*_ = −593 mV, *I*_*t*_ = 860 pA). (b) Amorphous short-range Pt-SMON in Ag(111) (*V*_*t*_ = −1489 mV, *I*_*t*_ = 140 pA). (c) High-resolution image showing the double (blue circle), triple (green circle) and cluster (yellow circle) coordination between N and Pt atoms (*V*_*t*_ = −969 mV, *I*_*t*_ = 450 pA). (d) Disruption of the amorphous short-range Pt-MOF (*V*_*t*_ = −599 mV, *I*_*t*_ = 1.11 nA) after annealing at 400 K.

In a similar experiment, we varied the order of deposition and the amount of precursor. First, we deposited Pt for 4 min, followed by the deposition of TPyPPB at RT, leading to similar results as for the sample with lower Pt coverage (see Supporting Information (SI), Figure S1a). Upon annealing at 400 K, this packing was destroyed, and the molecules tended to reorganize in a nonspecific manner concerning the coordination centers (SI, Figure S1b). Finally, we investigated the influence of the deposition sequence in forming the supramolecular nanostructures. First, TPyPPB molecules were deposited onto the clean Ag(111) surface at RT, followed by the deposition of Pt for 3 min at two different substrate temperatures. At 350 K, the sample displays the coexistence of the triangular packing and Pt islands decorated with TPyPPB molecules, as shown in Figure S1c. However, at *T*_s_ = 400 K, a unique structure was observed with a significant decrease in the Pt island size, allowing the TPyPPB molecules to coordinate with each other via the Pt atom/cluster (insert Figure S1d).^62^ In some cases, we can see three molecules linked to what appears to be a single Pt atom (or very small Pt cluster), as shown in the lower box of Figure S1d in the Supporting Information.

### Coordination Route 2: TPyPPB + Cl_2_PhPt

From the previous route, it is possible to conclude that an organometallic coordination is feasible through the N–Pt–N coordination bonds. However, it can occur in multiple configurations, such as two-, three-, and 4-fold coordination. To prevent Pt aggregation, it is necessary to stabilize individual Pt atoms on the Ag(111) surface, adjust the electronic structure of the Pt sites through the metal–support interaction, and improve the coordination environment.^63^ We thus establish another method for uniformly supplying Pt atoms or nanoparticles, that is, through immobilization using metal–organic ligands.^15,40,64–67^ To achieve this, we propose using a second molecular precursor that donates the Pt atom, dichloro­(1,10-phenanthroline) platinum­(II) (Cl_2_PhPt). The Cl_2_PhPt molecular precursor was selected based on previous studies, which demonstrate that organic linkers in MOFs containing nonmetal atoms like O, N, S, and halogen atoms (I, Cl) act as effective anchoring sites for robust coordination with isolated metal atoms (SA).^29,30,40,68^

First, Cl_2_PhPt was deposited, followed by the deposition of the TPyPPB on the Ag(111) surface at RT. Both molecular precursors were deposited at submonolayer coverages, allowing for molecular diffusion to occur. Figure S2 in the Supporting Information shows the coexistence of these two molecules on the surface. Cl_2_PhPt forms a self-assembled structure on the Ag(111) surface, as shown in Figure S2a. It happens in a similar way to other reported results for similar molecules.^69^ TPyPPB appears to be distributed without an apparent established order (SI, Figure S2b). Adopting the inverse order of deposition, first TPyPPB and subsequently Cl_2_PhPt, we can observe regions with well-ordered triangular patterns (as expected) and regions with the coexistence of TPyPPB and Cl_2_PhPt. However, the measurements also indicate a much higher level of mobile species at the surface. Notwithstanding, the results after heating treatment do not seem to depend significantly on the deposition order.

The molecular diffusion of precursors for SMON formation and the chemical activation of these molecular precursors (i.e., dehalogenation of Cl_2_PhPt) were studied during thermal treatment. Upon annealing the sample at 400 K for 30 min, we observed that the chemical interactions between the molecules evolved into a more complex configuration, resulting in various coordination motifs and arrangements, as seen in Figure 4. Figure 4a shows TPyPPB molecules forming different types of pores in this network, depending on the number of PhPt complexes involved in the coordination. The observed pore motifs are represented in the schematics of Figure 4b. We observe that some arms of TPyPPB are already interconnected head-on, while others are terminated by a small structure that is associated with the PhPt complex. The motifs in Figure 4b, 0—means no PhPt complex at the termination of the pyridyl group, while 1 up to 6 means we have 1 up to 6 PhPt complexes at the pyridyl terminations. Thus, annealing at 400 K promotes dehalogenation of the precursor (Cl_2_PhPt), which majority transforms Cl_2_PhPt into a new molecular complex, PhPt, maintaining the Pt atom in its structure, as indicated in the inset at the upper-right corner of Figure 4c. Nevertheless, at 400 K and for short annealing periods, a minority number of Pt–N bonds also start to break, as the formation of direct head-on interactions between the pyridyl groups starts to coexist with pyridyl-PhPt complexes. Dehalogenation is the primary step that occurs in halogenated precursors used in the Ullmann coupling reaction, and is well reported in the literature.^70^

**Figure 4:**
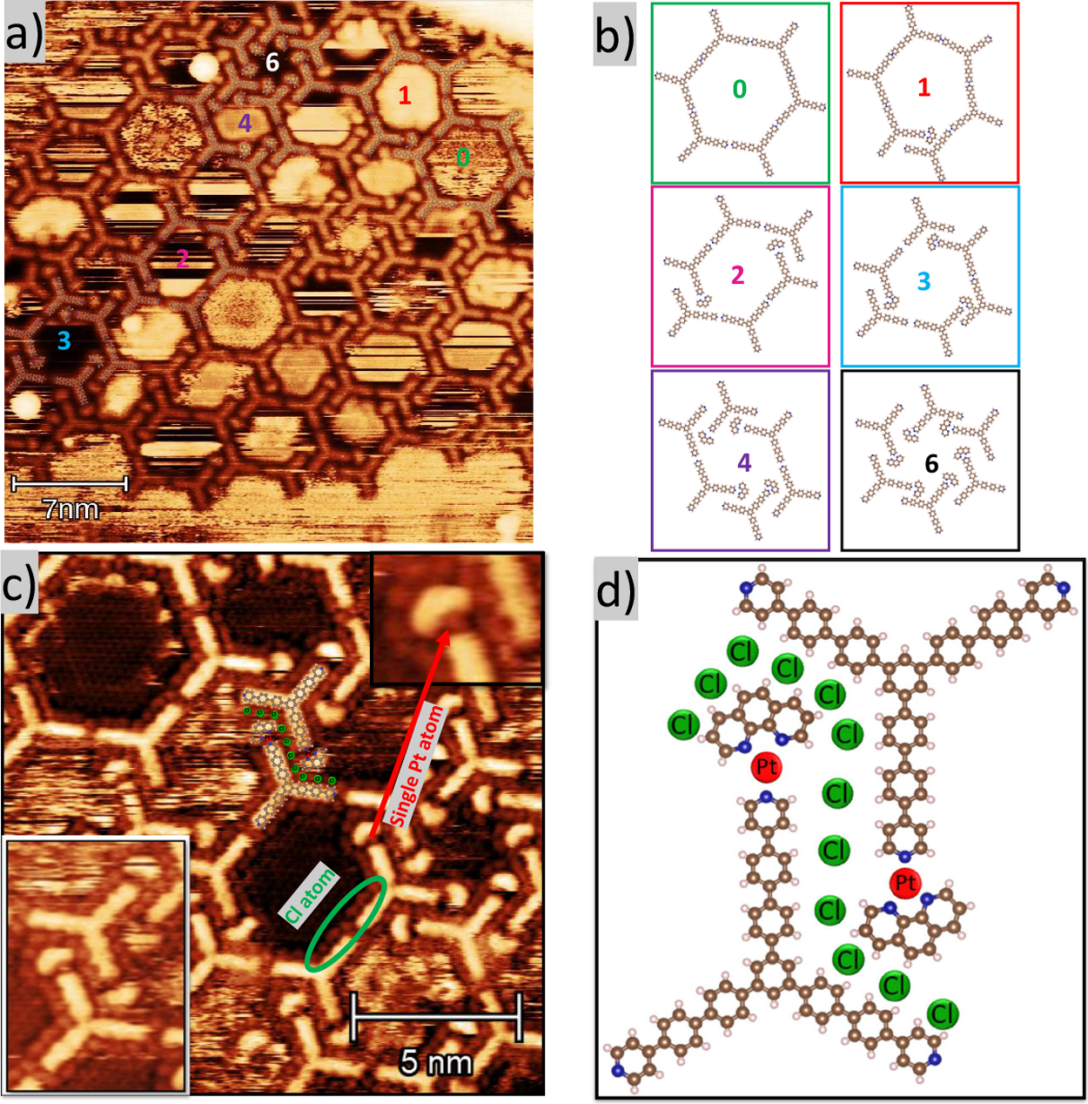
(a) STM image acquired after depositing the TPyPPB and Cl_2_PhPt precursors on the Ag(111) surface, followed by heating at 400 K for 30 min, showing the different types of porous structures formed (*V*_*t*_ = −1201.1 mV, *I*_*t*_ = 690 pA. (b) Schematic of different coordination motifs of TPyPPB and PhPt. The number inside each pore indicates the amount of PhPt required to perform the motifs. (c) High-resolution image showing Cl adatoms surrounding the molecular precursors. An enlarged region is shown in the upper-right box to facilitate the visualization of N–Pt coordination, while the lower-left box shows the interaction with Cl atoms. (d) Schematic molecular model. Green dots represent Cl adatoms interacting with the arms of the TPyPPB molecule, while red dots represent Pt–N coordination (*V*_*t*_ = – 423 mV, *I*_*t*_ = 1.14 nA).

The Cl atoms surround both molecular precursors, as shown in the Figure 4c. According to the literature, the dissociation of the (C–Cl) bond starts around 400 K and is nearly complete for *T* ≥ 450 K on Ag(111).^52,71–73^ When the Cl adatoms are placed along the arms of the TPyPPB and PhPt precursors, as shown in the inset at the lower left corner of Figure 4c, the molecules can weakly interact laterally via H···Cl···H bonds. At the same time, the Pt atom (indicated by red arrow in Figure 4c) maintains a N–Pt–N bonding structure. The schematic in Figure 4d shows the Pt atom (red ball) located at the end of the pyridyl group and the Cl atoms (green balls) surrounding both the TPyPPB molecules and the PhPt complex. This is because metallic platinum is characterized by its numerous stable oxidation states, flexible coordination geometries, and variable ligand exchange kinetics.^74,75^ These properties, combined with the ability of nitrogen-doped organic precursors to act as anchoring centers to immobilize individual metal atoms, facilitate the formation of stable coordination environments for Pt atoms.^29,30,40^ The sample after annealing 30 min at 400 K represents an intermediate state, showing the coexistence of metal (M) coordination of the pyridyl groups (i.e., N–M–N) in a head-on configuration, together with several PhPt complexes anchored to the remaining pyridyl group of TPyPPB. Since Ag adatoms can not coordinate to the pyridyl group, as previously reported,^52,54^ the metal center in the N–M–N coordination must be a single Pt atom.

The phase shown in Figure 4 is an intermediate and not entirely stable one. In a series of consecutive STM images, we observed that the molecules moved continuously, entering and exiting the pores. The network itself also exhibits dynamic behavior, disassembling and reassembling (see Video 1 in the SI). To drive the system to the final coordination configuration, a long annealing process is applied. After a 2 h annealing at 400 K, the TPyPPB molecules begin to form hexagonal networks connected by Pt atoms, which are in a 4-fold coordination by two N atoms of the TPyPPB and two Cl atoms, as shown in Figure 5. This means that two adjacent TPyPPB molecules align collinearly (head-on) and bind to each other by their pyridyl end groups. Due to the repulsive force between nitrogen atoms, we propose a linkage through a single Pt adatom. To stabilize the structure, the Pt atom also coordinates with two Cl atoms, forming a 4-fold coordination, as shown in the high-resolution STM image of Figure 5c, and illustrated in the model of Figure 5d. This arrangement indicates that chlorine is responsible for the stabilization of the structure with a single Pt atom, Pt­(N_2_Cl_2_) coordination center, to form the SMONs.^76,77^ Compared with the arrangement in Figure 3b, we can infer that the presence of Cl atoms coordinating with Pt may be responsible for the formation of the hexagonal supramolecular structure. Literature has shown the importance of halogens in surface synthesis; for example, Ceccatto et al.^52^ investigated the formation of highly ordered 2D porous supramolecular networks of TPyPPB on Ag(111) and its modification by deposited chlorine. In that case, the porous nanostructure is characterized by regular triangular pores and is stabilized by N···H hydrogen bonds between the TPyPPB molecules. The structure of the network can be modified by depositing Cl, where TPyPPB forms two distinct new phases, namely a mixed and an inverted phase.^52^ Xie et al. demonstrated that I atoms in surface synthesis induce morphological transformations in 2D supramolecular networks.^78^

**Figure 5:**
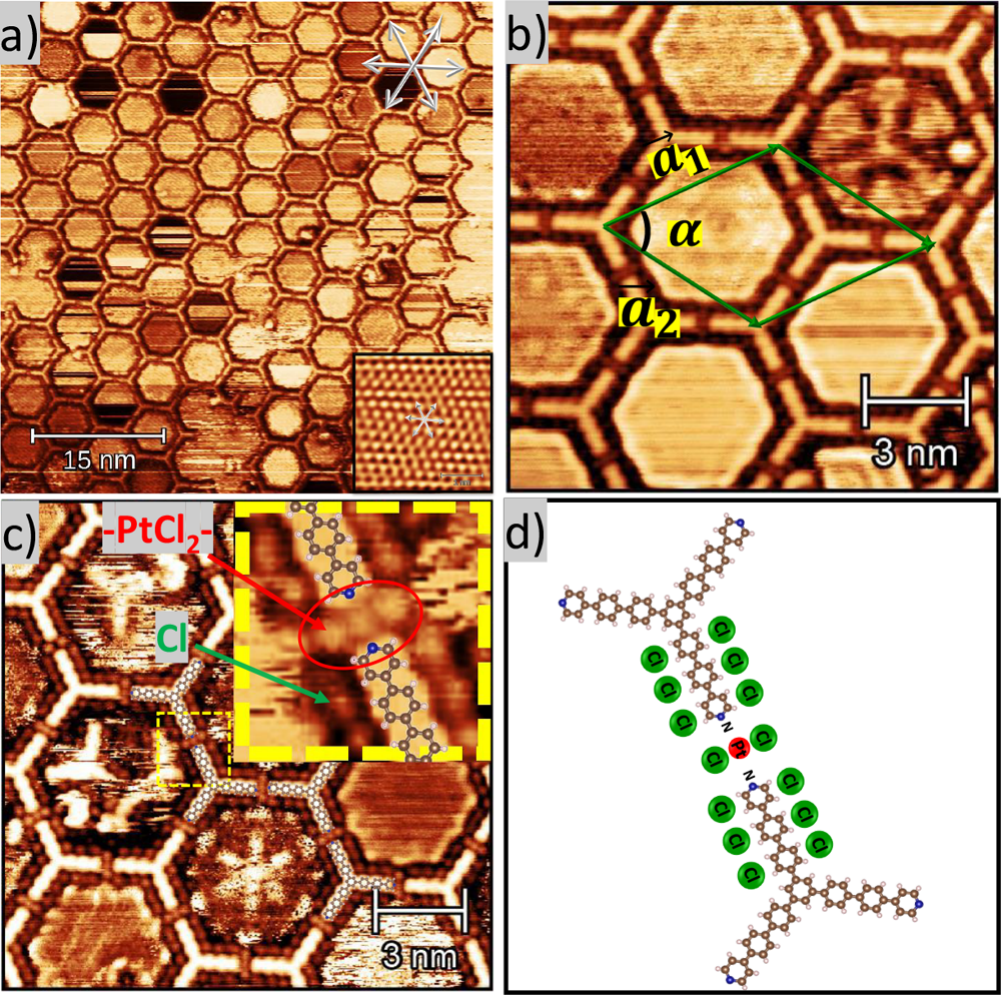
STM images after depositing the TPyPPB and Cl_2_PhPt precursors on the Ag(111) surface, followed by heating at 400 K for 2 h. (a) STM image with the main crystallographic directions of the Ag(111) surface highlighted in green at the top of the image. The legs of the TPyPPB molecule are oriented along the crystallographic directions of the substrate (*V*_*t*_ = −758 mV, *I*_*t*_ = 340 pA). (b) STM image with the lattice vectors $|{\vec{a}}_{1}|=|{\vec{a}}_{2}|=(5.5\pm 0.3)\;\mathrm{nm}$ highlighted in green (*V*_*t*_ = −436 mV, *I*_*t*_ = 530 pA). (c) High-resolution STM images. The TPyPPB molecular structure superimposed, showing the intermolecular interactions through the coordination of a single Pt atom with 2 N atoms and 2 Cl atoms (*V*_*t*_ = −911 mV, *I*_*t*_ = 490 pA). (d) Schematic molecular model.

The lattice parameter of the hexagonal porous network shown in Figure 5b was determined as $|{\vec{a}}_{1}|=|{\vec{a}}_{2}|=(5.5\pm 0.3)\;\mathrm{nm}$ and α = 60°. They are rotated by 30° concerning the Ag(111) main crystallographic direction, thus forming a superstructure of $(11\sqrt{3}\times 11\sqrt{3})R30^{{}^{\circ}}$ . These values were obtained by directly measuring the lengths of the vectors from several STM images. The unit cell contains a total of three Pt atoms and two TPyPPB molecules. Furthermore, by using atomic resolution measurements of the Ag(111) surface (inset of Figure 5a), we can determine that the arms of the TPyPPB molecule are oriented along the main crystallographic directions.

Through STM images with atomic resolution, it can be observed that in some of the pores of our nanostructures, both atoms and molecules are confined. Figures 5c and 6a shows the pore-forming TPyPPB molecule and PhPt complex inside a hexagonal pore, where the TPyPPB molecule has three PhPt complexes linked to the end pyridyl groups. This new larger molecule formed inside the pore exhibits behavior similar to that of free molecules, acting as rotors (triangular “propellers”) that are stabilized only in positions where a coordination point is present (see schematics in Figure 6b). This behavior was observed through a series of consecutive STM images, which revealed the dynamics of these molecules within the pore (see Figure S4 and Video 2 in the SI). In literature, Kühne et al. studied a similar dynamics of guest molecules trapped in the hexagonal pores of surface metal–organic networks (SMONs).^79^ Variable-temperature STM revealed that the trimolecular guest units underwent rotational motion, preserving chirality within the pores. In another study, Zhang et al. reported confinement of Bi nanoclusters using SMONs pores with two different sizes.^80^ The Cu-TPyB and Cu-ext-TPyB SNOMs have pores with internal diameters of 1.9 and 3.4 nm, respectively. In the Cu-TPyB SMON, the Bi clusters were very uniform in size. High-resolution images showed that the Bi clusters filled the hexagonal pores. The model suggests that the maximum number of Bi atoms in a cluster is 19 when the Bi atoms are closely packed. In the Cu-ext-TPyB SMON, the clusters were less uniform than those in the Cu-TPyB pores. The model suggests that in the larger pore, a cluster may contain up to 61 Bi atoms.

**Figure 6:**
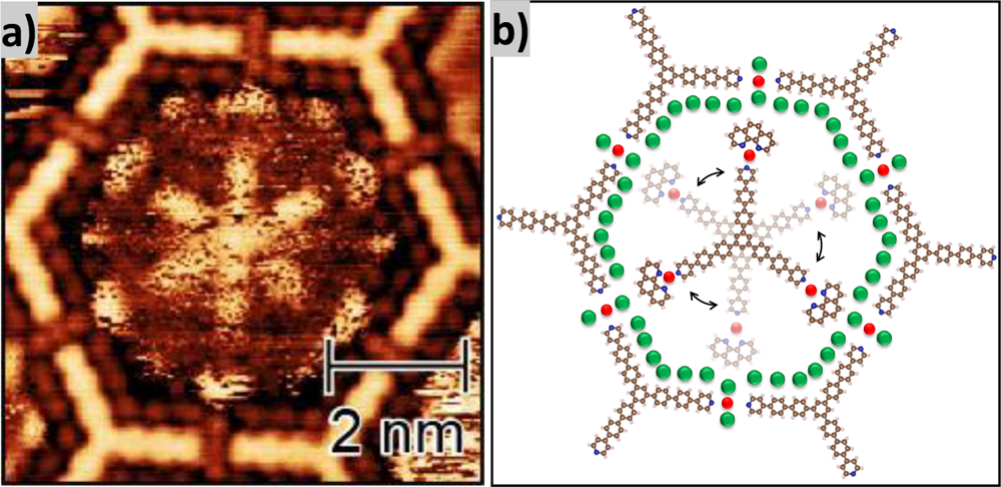
High-resolution STM images showing the trapping of molecules within the hexagonal pores of the network. (a) Larger molecule, formed by TPyPPB linked to PhPt complexes, rotates within the pores and is stabilized only at points where there is an N–Pt–Cl coordination (*V*_*t*_ = −911 mV, *I*_*t*_ = 490 pA). (b) Schematic molecular model. Green dots represent Cl adatoms interacting with the arms of the TPyPPB molecule, while red dots represent Pt atoms.

In this study, we have elucidated the remarkable properties of exceptionally large porous nanostructures, which exhibit the capacity to establish an advantageous environment for the adsorption and reaction of molecules within confined spaces (i.e., “host–guest” chemistry). The uniform distribution of pores, along with their dimensions and geometrical configurations, coupled with the tailored chemical characteristics of the nanopores (i.e., single Pt atoms at specific positions and valence state), might create binding sites for guest molecules or atoms. Such a configuration might induce selective binding, for instance, targeting molecules of particular sizes (e.g., 5.5 nm) or facilitating the assembly of uniquely shaped polymers, such as triangular rotors. Overall, the versatility and functionalization potential of 2D porous nanostructures, combined with STM direct visualization, make them a valuable tool for modeling the physical-chemical mechanisms involved in “host–guest” chemistry.

## Conclusions

In summary, we present a study on the synthesis of 2D porous metal–organic networks (Pt-SMONs) from the molecular precursor TPyPPB on the Ag(111) surface. Two different deposition methods yield distinct results, with one being more effective. In the first method, Pt atoms are deposited directly onto the Ag(111) surface. Depending on the thermal treatments applied through annealing processes and the variation in Pt atom concentration, a porous network can be formed, coordinated by either a single Pt atom (with double or triple N–Pt coordination) or by multiple Pt atoms. However, this network has limited extension and a high number of defects due to the high free energy of Pt atoms, which favors the formation of Pt islands on the Ag(111) surface. This hinders the interaction between TPyPPB molecules and Pt atoms, thus preventing the stabilization of the metal–organic network. On the other hand, this network can be modified and stabilized using a second molecular precursor, Cl_2_PhPt, which incorporates the coordinated Pt atom into its structure. In this second method, a honeycomb-like nanostructure is obtained. At room temperature, the two molecular precursors act independently; however, after annealing treatments at 400 K, the system transforms into more complex structures, ultimately forming a hexagonal porous metal–organic network.

The Cl_2_PhPt precursor, after an initial annealing process, forms a new dechlorinated PhPt complex that remains on the surface. The TPyPPB molecules interact with Cl atoms, which are located around the “arms” of the molecule, forming C–H···Cl bonds, as well as with the remaining dechlorinated complex through its Pt atom, establishing N–Pt bonds. This results in an amorphous network. By increasing the annealing time, the nanostructure is stabilized, ultimately forming a 2D hexagonal metal–organic network. In this process, phenanthroline desorbs from the surface, exposing the Pt atom, which establishes a 4-fold coordination with two Cl atoms and two N atoms. This nanostructure has regular hexagonal pores with an area of ∼26 nm^2^ functioning as a suitable matrix for the confinement of molecules as large as 5.5 nm in diameter.

## Supplementary Material







## Notes

Additional experimental results, including STM images for the two different synthesis route of Pt-SMONs on Ag(111) (PDF)
Mobile phase with molecules entering and exiting the pores and a dynamic behavior of the Pt-SMONs, disassembling and reassembling (GIF)
Propellers (TPyPPB + PhPt complex) rotating inside the pores (GIF)
