# Supplementary material for: Tailoring Pt-Based Organometallic Porous Network on Ag(111): A Model System for “Host-Guest” Chemistry
Source: ACS Nanosci Au. 2025 Nov 7;6(1):139–47. doi: 10.1021/acsnanoscienceau.5c00124 (PMC12921605; doi:10.1021/acsnanoscienceau.5c00124)
Supplement: Supplementary file 1 [file ng5c00124_si_001.pdf]

Supporting Information for: Tailoring Pt-based Organometallic Porous Network on Ag(111): a Model System for "Host-Guest" Chemistry

Vanessa Carreño-Diaz,[*,†] Alisson Ceccatto,[†,‡] Eidsa Brenda da Costa Ferreira,[†] Majid Shaker,[‡] Hans-Peter Steinrück,[‡] and Abner de Siervo[*,†]

†*Instituto de Física Gleb Wataghin Universidade Estadual de Campinas UNICAMP, 13083-970 Campinas, Brazil*

‡*Lehrstuhl für Physikalische Chemie II, Friedrich-Alexander-Universität Erlangen-Nürnberg, Egerlandstr. 3, 91058 Erlangen, Germany*

E-mail: carrenod@ifi.unicamp.br; asiervo@ifi.unicamp.br

Additional results

This supporting information shows additional STM figures of the 2 different route on Ag(111).

Coordination Route 1: TPyPPB + Pt metal. Coordination Route 2: TPyPPB + Cl_2PhPt.

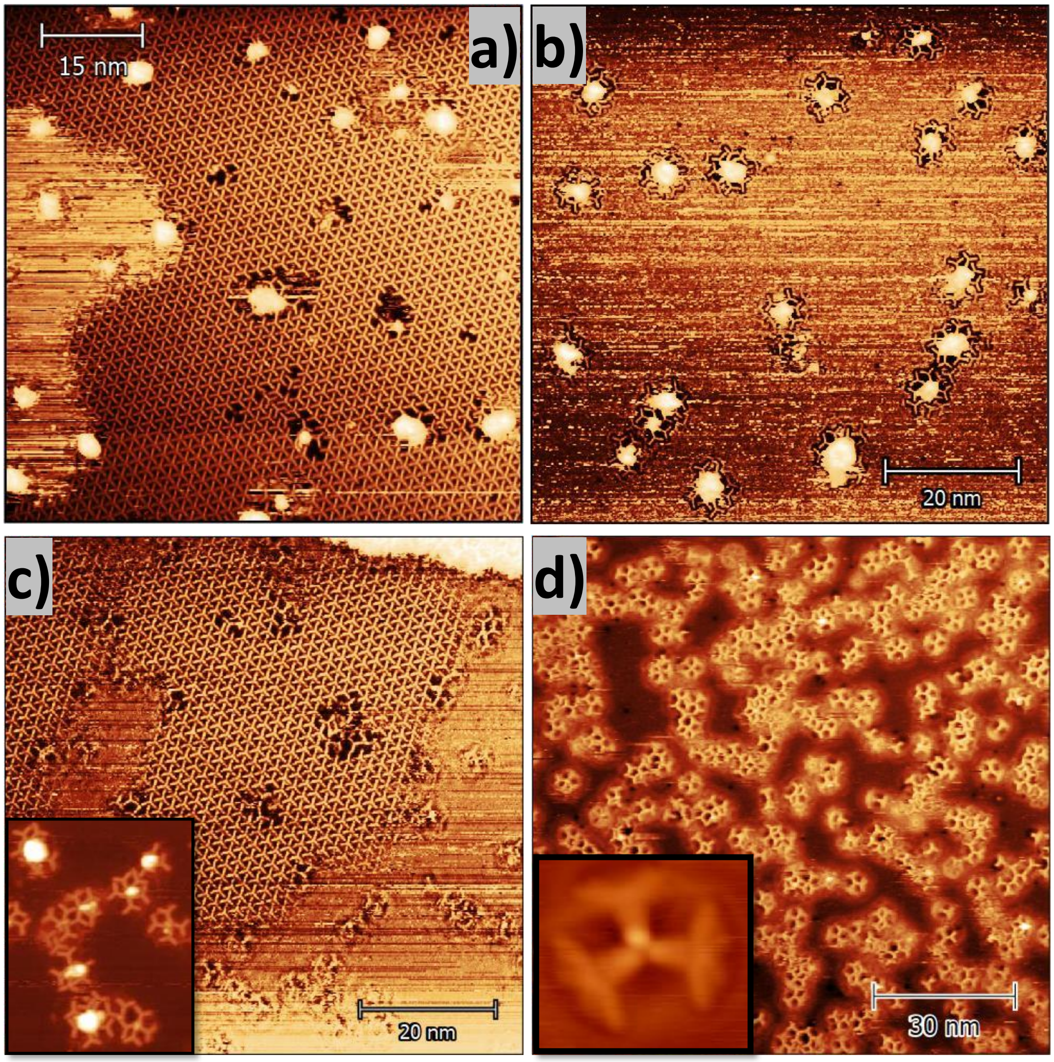

Figure S1: STM image of Pt deposition (4 min) followed by TPyPPB molecules (30 min) on Ag(111): (a) At room temperature – triangular packing and Pt island formation. (b) After annealing at 400 K – disruption of the triangular packing ($V_t = -1030\ mV, I_t = 350\ pA$). STM image of TPyPPB molecules (30 min) deposited at room temperature, followed by Pt deposition (3 min) on Ag(111) after heating: (c) $T_s = 350\ K$ – triangular packing and region with multiple arrangements and coordination types with Pt island (lower box) ($V_t = -940\ mV, I_t = 500\ pA$). d) $T_s = 400\ K$ ($V_t = -593\ mV, I_t = 1.11\ nA$) lower box detailed view of a triple coordination with a single Pt atom (or very small Pt cluster) ($V_t = -969\ mV, I_t = 230\ pA$).

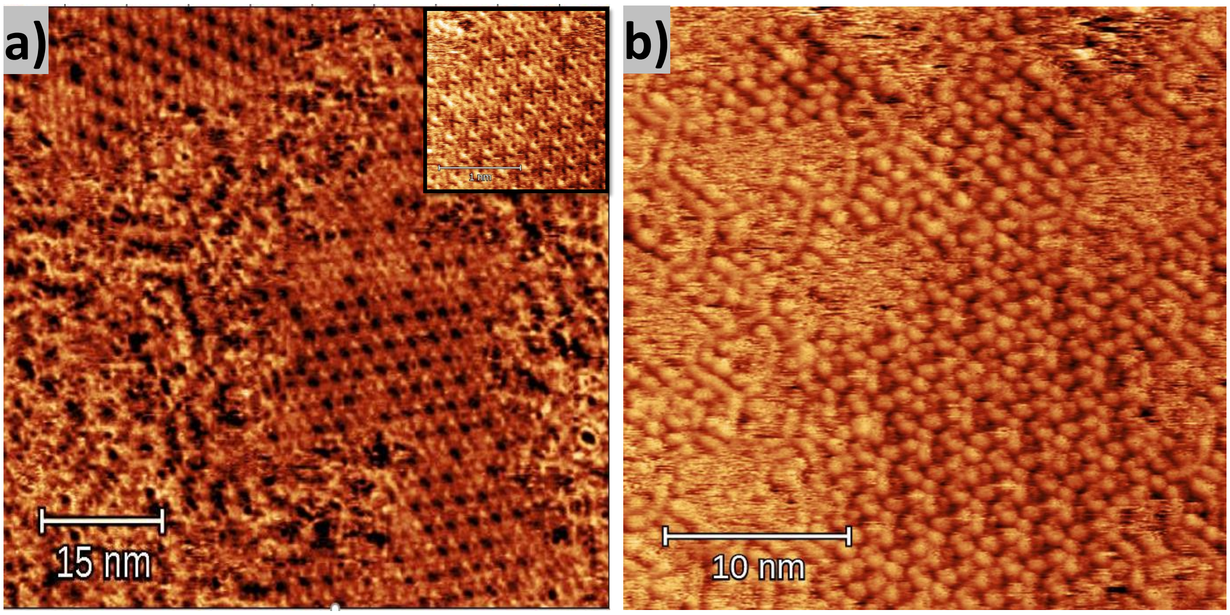

Figure S2: STM images acquired in constant current mode: (a) Self-assembly formed by Cl_2PhPt molecules on the Ag(111) surface at room temperature. An enlarged region is shown in the upper box to facilitate visualization ($V_t = -363\ mV, I_t = 660\ pA$). (b) TPyPPB molecule dispersed without any apparent order among the Cl_2PhPt molecules.

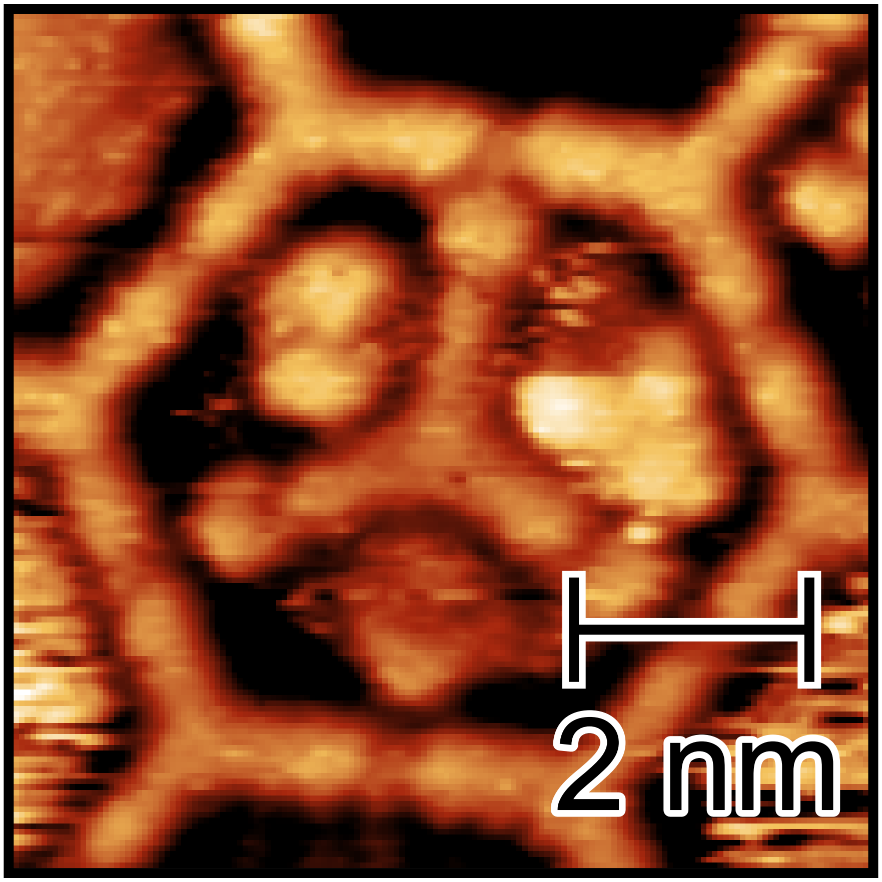

Figure S3: STM image acquired in constant current mode showing the TPyPPB-PhPt new derivative and Cl_2PhPt trapped inside the hexagonal pore. ($V_t = -436\ mV, I_t = 890\ pA$).

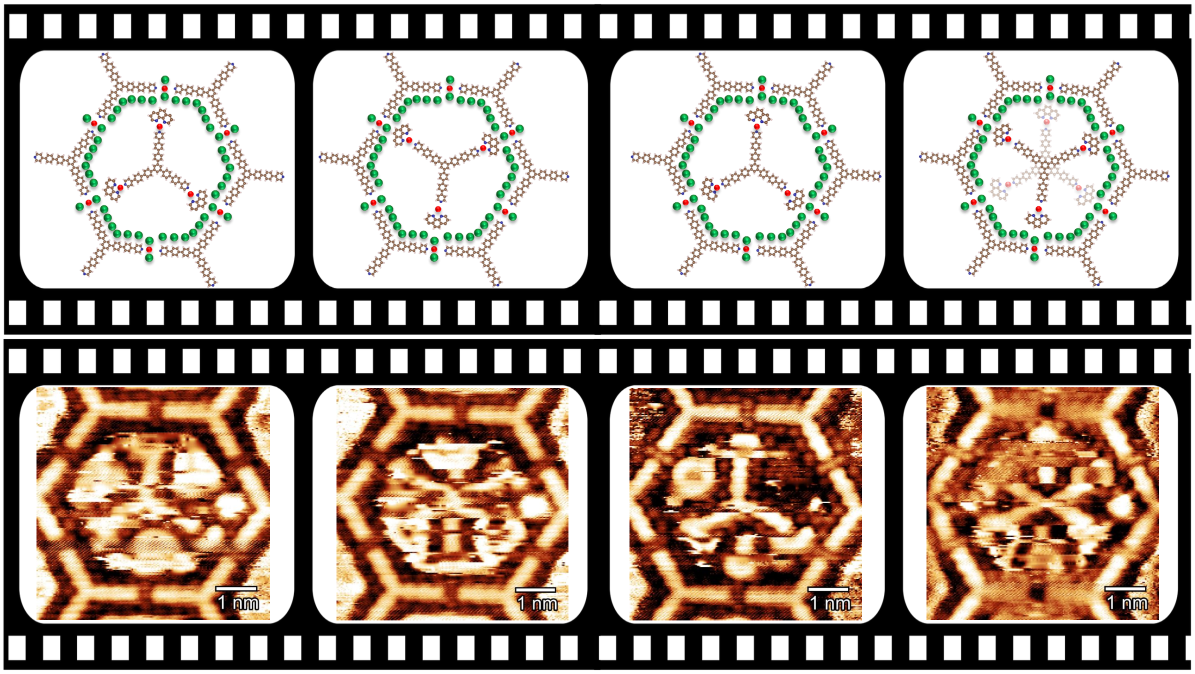

Figure S4: (upper panel) Schematic representation and (lower panel) consecutive STM images taken from (Video 1) showing the rotation of molecules within a pore of the network. ($V_t = -436\ mV, I_t = 890\ pA$).

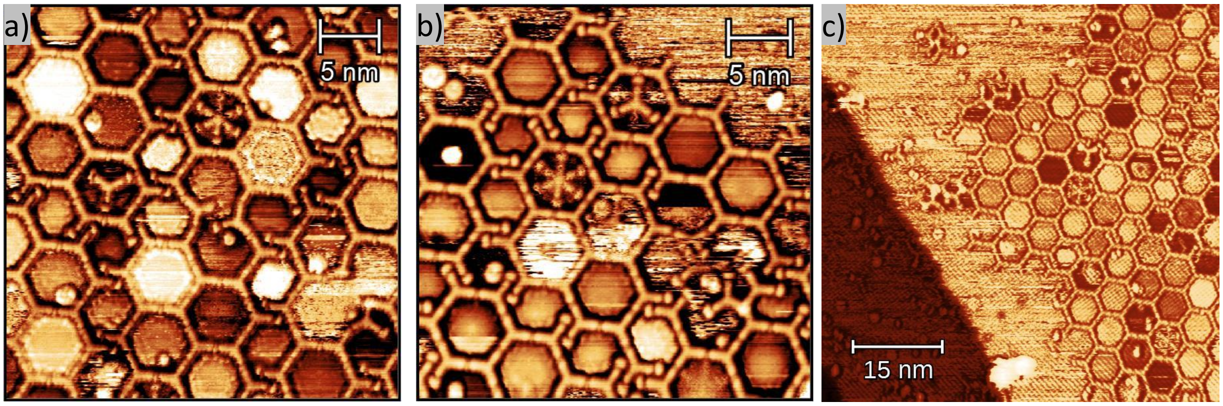

Figure S5: STM images showing a hexagonal porous network hosting guest molecules confined within the pores a)($V_t = -883\ mV, I_t = 1150\ pA$) b) ($V_t = -969\ mV, I_t = 420\ pA$) c) ($V_t = -1316\ mV, I_t = 1190\ pA$). .
