# Supplementary figures and images for: Tailoring Pt-Based Organometallic Porous Network on Ag(111): A Model System for “Host-Guest” Chemistry
Source: ACS Nanosci Au. 2025 Nov 7;6(1):139–47. doi: 10.1021/acsnanoscienceau.5c00124 (PMC12921605; doi:10.1021/acsnanoscienceau.5c00124)

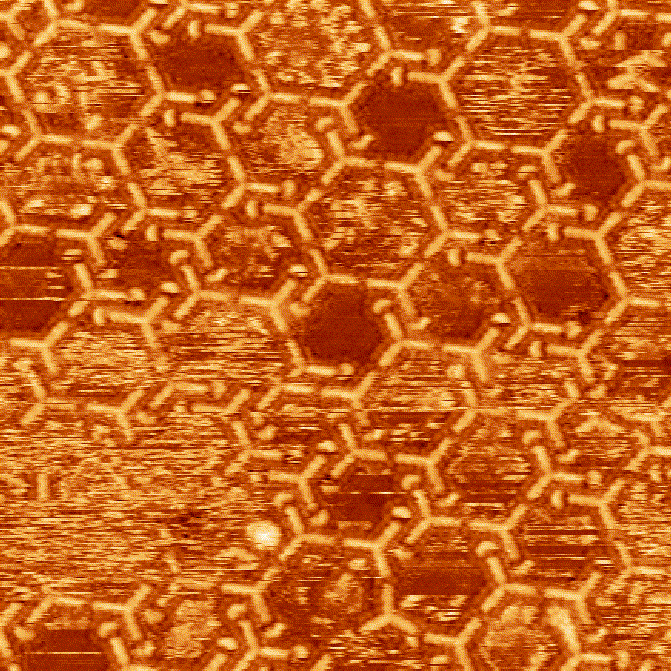

Supplement: Supplementary file 2 [file ng5c00124_si_004.gif]

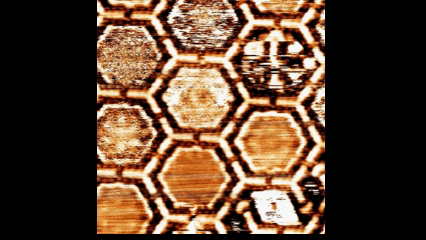

Supplement: Supplementary file 3 [file ng5c00124_si_005.gif]
